# Fatal thoracic aortic aneurysm and dissection in a large family with a novel MYLK gene mutation: delineation of the clinical phenotype

**DOI:** 10.1186/s13023-018-0769-7

**Published:** 2018-03-15

**Authors:** Adel Shalata, Mohammad Mahroom, Dianna M. Milewicz, Gong Limin, Fadi Kassum, Khader Badarna, Nader Tarabeih, Nimmer Assy, Rona Fell, Hector Cohen, Munir Nashashibi, Alejandro Livoff, Muhammad Azab, George Habib, Dan Geiger, Omer Weissbrod, William Nseir

**Affiliations:** 1 Simon Winter Institute for Human Genetics, B’nai Zion Medical Center, P.O.B 4940, 31048 Haifa, Israel; 2 Genetic Unit, Ziv Medical Center, Safed, Israel; 3 Ginatuna Association, Sakhnin, Israel; 4 Department of Internal Medicine, McGoven Medical School, University of Texas Health Science Center at Houston, Houston, USA; 5 Department of Internal Medicine, Western Galilee Medical Center, Nahariya, Israel; 6 Research Unit, Western Galilee Medical Center, Nahariya, Israel; 7 Department of Pathology, Western Galilee Medical Center, Nahariya, Israel; 8 Department of Pathology, Laniado hospital, Netanya, Israel; 9 Faculty of medicine, Technion, Haifa, Israel; 10 Rheumatology unit, Laniado hospital, Netanya, Israel; 11 Computer Science Department, Technion – Israel Institute of Technology, Haifa, Israel; 12 Department of Internal Medicine, EMMS Nazareth Hospital, Nazareth, Israel

**Keywords:** MYLK gene mutation, Aortic aneurysm and dissection, Genotype-phenotype

## Abstract

**Background:**

Thoracic and abdominal aortic aneurysms and dissection often develop in hypertensive elderly patients. At higher risk are smokers and those who have a family history of aortic aneurysms. In most affected families, the aortic aneurysms and dissection is inherited in an autosomal dominant manner with decreased penetrance and variable expressivity. Mutations at two chromosomal loci, TAA1 at 11q23 and the TAA2 at 5q13–14, and eight genes, *MYLK*, *MYH11*, *TGFBR2*, *TGFBR1*, *ACTA2*, *SMAD3*, *TGFB2*, and *MAT2A*, have been identified as being responsible for the disease in 23% of affected families.

**Results:**

Herein, we inform on the clinical, genetic and pathological characteristics of nine living and deceased members of a large consanguineous Arab family with thoracic aortic aneurysm and dissection who carry a missense mutation c.4471G > T (Ala1491Ser), in exon 27 of *MYLK* gene. We show a reduced kinase activity of the Ala1491Ser protein compared to wildtype protein. This mutation is expressed as aortic aneurysm and dissection in one of two distinct phenotypes. A severe fatal and early onset symptom in homozygous or mild late onset in heterozygous genotypes.

**Conclusions:**

We found that MYLK gene Ala1491Ser mutation affect the kinase activity and clinically, it presents with vascular aneurysms and dissection. We describe a distinct genotype phenotype correlation where; heterozygous patients have mild late onset and incomplete penetrance disease compared with the early onset severe and generally fatal outcome in homozygous patients.

## Background

Thoracic aortic aneurysms and aortic dissections (TAAD) often develop in hypertensive individuals, predominantly in men aged 65 years and older who may have a family history of aortic disease. In most affected families, a predisposition for TAAD is inherited in an autosomal dominant manner with decreased penetrance and variable expressivity. The prevalence of thoracic aortic aneurysms is underestimated because these aneurysms are typically asymptomatic. Nevertheless, the occurrence of aortic aneurysms has gradually increased over the past few decades [1–4]. The estimated annual incidence of rupture of thoracic aortic aneurysms (TAA) is 3/100000 persons and 9/100000 persons for abdominal aortic aneurysms (AAA) [1, 5]. In the United States, rupture of either a TAA or an AAA is responsible for 15,000 and nearly 6000 deaths annually, respectively [1, 6].

The results of several familial aggregation studies indicate that as many as 20% of all patients with a non-syndromic TAAD have a first-degree relative with a history of TAAD [7, 8]. In most affected families, TAAD is inherited in an autosomal dominant manner with decreased penetrance and variable expressivity [9]. Mutations in 13 genes, *MYLK*, *MYH11*, *TGFBR2*, *TGFBR1*, *ACTA2*, *SMAD3*, *TGFB2*, *TGFB3*, *PRKG1*, *FOXE3*, *MFAP5*, *FBN1*, and *MAT2A*, have been identified as being responsible for the disease in approximately 30% of affected families [9–13].

Few MYLK gene mutations were identified as co-segregating with the disease in families with FTAAD including c.5275 T > C, p.S1759P; c.5260G > A, p.A1754T; c.3637G > A, p.V1213 M; c.4195G > A, p.E1399K; c.4438C > T, p.G1480X [12]; and the mutation c3272_3273del, p.S1091X described recently [14]. Moreover, novel mutations in MYLK were identified in recessive Megacystis Microcolon Intestinal Hypoperistalsis Syndrome. MMIHS is a congenital disorder characterized by loss of smooth muscle contraction in the bladder and intestine [15].

In the current study, the clinical, genetic, and pathological characteristics of 9 living and deceased members of a large consanguineous Arab family with a TAAD are reported.

## Methods

The study included 9 patients (5 living and 4 deceased) of a large consanguineous Arab family with thoracic aortic aneurysm and dissection and their family members (Tables 1 and 2). Institutional review board (IRB) approval was obtained and written consent was provided from all study participants. We interviewed and examined the 5 living patients and reviewed the medical and post-mortem records of the 4 deceased patients. In order to complete the clinical picture, we also interviewed and reviewed the medical records of the family members (spouse, brothers, sisters, and parents) of the 9 patients.

**Table 1:** Patients clinical presentation

Patient ID	Gender	Age	Genotype	Clinical presentation						
				Pain				Dyspnea	limbs numbness and coldness	Watery diarrhea
				Chest	Abdomen	Limbs				
						U	L			
V-2	M	33	Hmz*	+	+	+	+	+	+	+
VI-27	F	33	Hmz	+	+	+	+	+	+	+
VI-26	F	42	Hmz	+	+	+	+	+	+	–
VII-1	M	34	Hmz	+	+	+	+	+	–	+
VII-3	M	33	Hmz	+	+	+	+	+	+	+
VI-23	M	34	Hmz*	+	+	+	+	+	+	+
Av.		34.83 (+ 7, −1.8)								
Group-II Patients										
V-15	M	69	Het.	+	+	–	+	+	+	–
V-22	M	69	Het.	+	–	+	–	+	–	–
VI-24	M	54	Het.	+	–	–	–	+	–	–
Av.		64 (+ 5, −10)								

**Fig. 1:**
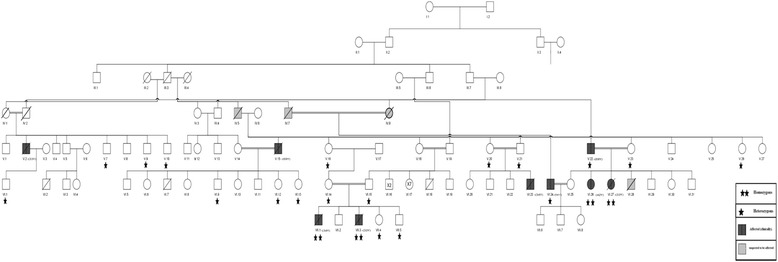
The family pedigree of a consanguineous multigenerational family with thoracic and abdominal aortic aneurysm and dissection TAAD. Definition: black fill shape- affected (clinically). Gray fill shape: most probably affected according to the clinical picture. Single (*) or two (**) asterisks, heterozygous or homozygous for the mutation

Detailed clinical data for the affected individuals from group-I (VI-5, VII-1, VII-3, VI-23, VI-26 and VI-27) and group-II (V-22, VI-24 and V-15) (Figure-1) are summarized in Tables 1 and 2

*M* male, *F* female, *U* Upper, *L* Lower, *Av* Average, *R.D* Recurrent Dissection, *Hmz* homozygous, Hmz* the homozygous state was inferred, *Het* Heterozygous

**Table 2:** Vascular findings

Vascular Dissection & Aneurysm (mm)										
Patient ID	Asc. Aorta	Desc. Aorta	Abd. Aorta	Renal	Iliac	SMA	Aortic Arch/Root	Subclavian Dilatation	Innominate Dilatation	Outcome
Group-I Patients										
V-2	+	+	+	+	+	+	–	–	–	Deceased
VI-27	+	+	+	+	+	+	–	+ (Lft)	–	RD, deceased
VI-26	–	+	+	+	–	–	+ arch	+ (Rt)	+ (**15)	Observation
VII-1	–	+	+	–	+	+	–	–	–	Deceased
VII-3	+	+	+	+	+	+	–	+ (Rt) (15)	+ (16)	RD, deceased
VI-23	+	+	+	+	–	+	–	–	–	RD, deceased
Group-II Patients										
V-15	+	+	+	+	+	+	+ root	–	–	RD, deceased
V-22	–	+	–	–	–	–	+ (80) root	–	–	Observation
VI-24	+	+	–	–	–	–	+ (75) root	–	–	Observation

*Asc* Ascending, *Desc* Descending, *Abd* Abdomen, *SMA* Superior Mesenteric Artery. In the dissection and aneurysm columns data: All the “+”signs, except those in the Subclavian and Innominate columns, represent dissection. The numeric values in the parentheses represent the dilatation/aneurysm measurement (millimeter units). ** aneurysmatic dilatation (up to 15 mm) between the left subclavian and the carotid arteries

Histologic studies: Formalin-fixed and paraffin-embedded specimens of the excised aneurysms from the living and deceased patients, in whom an emergency surgical repair was performed, were examined under a Nikon light microscope (magnification X100) after eosin-hematoxylin staining. Immunohistochemical staining for myosin light chain kinase (MLCK): The formalin-fixed and paraffin-embedded specimens of the excised aortic aneurysms from the living and deceased patients and an unrelated patient of the same ethnic background were examined after immunohistochemical staining for MLCK. For this purpose, serial sections (5-μm thick) of each specimen were first deparaffinized and rehydrated in xylene (two 5-min changes) and a graded series of alcohol bath solutions (two 5-min changes in 100% ethanol; one 5-min change in 90% ethanol; one 5-min change in 70% ethanol). After two 5-min washings of the slides in tap water, the antigenic epitope was unmasked by incubating the slides in Tris-EDTA buffer pH 9.5 at 95 °C for 20 min, cooling them in a Coplin jar for 20 min at room temperature, washing them in running water for five minutes, and then washing them twice in phosphate buffered saline (PBS) for five minutes. Before immunohistochemical staining, endogenous peroxidase in each section was quenched by immersing the slides in 3% hydrogen peroxide in methanol for 20 min at room temperature, followed by three 5-min washings in PBS with 0.5% Tween. Immunohistochemical staining of the sections comprised an overnight incubation of the sections with a 1:100 monoclonal MLCK antibody (#2095–1, Epitomics) at 4 °C, and then exposing the incubated sections to the biotinylated secondary antibody and chromogen in the Histostain®-*Plus* Bulk Kit Zymed 2nd Generation LAB-SA Detection System (#859243, Invitrogen Corp., Camarillo, CA, USA) according to the manufacturer’s instructions. The slides were then examined under a Nikon light microscope (magnification X100).

The immunohistochemical staining was measured both by its intensity and its extent. Staining intensity ranged from 0 (weakest) to 3 (strongest). Staining extent (0%–100%) was converted to the scale 0 to 4 as follows: 0 (no staining), 1 (1%–5%), 2 (6%–25%),3 (26%–50%), or 4 (4 50%). The combined score is the sum of the 2 measures, and is thus on a scale of 0 to 7 [16].

### Whole genome genotyping and sequencing of MYLK

DNA was extracted from peripheral blood leukocytes of two patients, VII-3 and VI-26 (Fig. 1), using Qiagen’s QIAamp 96 DNA QIAcube HT Kit (#51331, Qiagen, Venlo, Netherlands) for whole genome genotyping to identify chromosomal regions with extended haplotype homozygosity. After purifying the DNA, whole genome genotyping was done using Illumina’s HumanCytoSNP-12v2.1 DNA Analysis BeadChip Kit (#WG320–2102; Illumina, CA, USA). According to the kit’s manufacturer, this array is a powerful whole-genome scanning panel that is designed for efficient high-throughput analysis of genetic and structural variations in the entire genome that are the most relevant to human disease, including the loci and genes which are known to be involved in TAA and TAAD [9–13]. Disease genes were also detected in the DNA samples using Superlink-Online SNP (http://cbl-hap.cs.technion.ac.il/superlink-snp/), an online system for exact and approximate genetic linkage of single nucleotide polymorphism (SNP) data in large pedigrees [17]. The exon sequences and the flanking intron sequences of *MYLK* were amplified by polymerase chain reaction (PCR), and the PCR products were sequenced by Sanger sequencing using the standard protocols. Finally, a segregation analysis to test for segregation of the rare variant in *MYLK* with disease in the family was done using DNA that were extracted from peripheral blood leukocytes of four more patients and 36 apparently healthy family members, and the paraffin-embedded aortic artery samples of two patients (Fig. 1, Table 1).

### Myosin light chain kinase activity assays

The plasmid of human full-length short form of myosin light chain kinase was obtained (Oragene) and inserted into a pCMV6-XL4 vector with additional amino acids (Ala-Gly-Ala-Gly-Ser-Thr-Val) followed by 3 FLAG tags at the amino-terminus. Oligonucleotide-directed mutagenesis was introduced to produce the mutation with A1491S (G > T). The construct was confirmed by restriction enzyme digestion and DNA sequencing. HeLa cells were transfected with the FLAG-MYLK constructs using lipofectamine 2000 transfection reagent (Invitrogen) and cell lysates harvested 48 h later. Wild type and mutant MLCK assessed by immunoblotting with anti-FLAG monoclonal antibody (M2, Sigma). The proteins were purified using ANTI-FLAG M2 Affinity Gel beads (Sigma). Kinase activity was determined based on P^32^ incorporation into the MYLK substrate MYL (Biomol). K_m_ and V_max_ values were obtained from non-linear regression program (Prism 5). Calmodulin activation was assessed by P32 incorporation into MYL in the presence of saturating concentration of CaCl_2_ and varying amounts of calmodulin. K_CaM_ values were assessed using non-linear regression program.

## Results

The pedigree of the 9 patients, who were members of a large consanguineous Arab family, is displayed in Fig. 1. The presence of aortic disease was confirmed in these individuals by computerized tomographic angiography (CTA), catheterization, visual identification of the aneurysm during surgery, and/or a post-mortem examination. The 9 patients could be subdivided into two groups, group I and group II, according to their medical history, the results of their clinical examination, homozygosity mapping analysis, and sequencing of *MYLK* (Tables 1 and 2).

### Group I patients

**Fig. 2:**
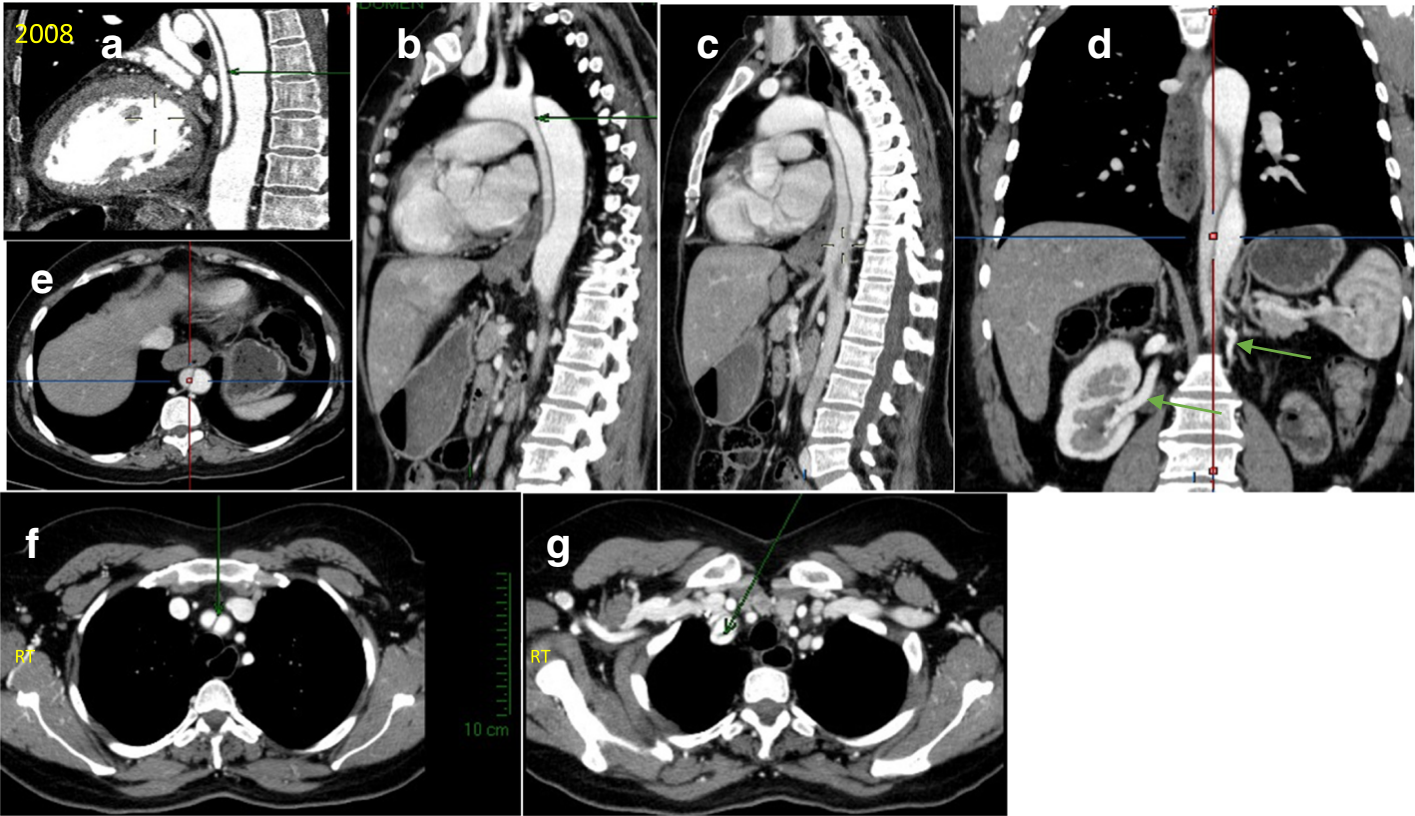
Computerized Tomography angiography- CTA of patient VI-26. CTA demonstrated Type-3B aortic dissection (Arrow, sagittal planes **a** (2008), **b**, **c**, (2011)) beginning distal to left subcalvian artery (LTSCA) that continued down 12 mm above to the bifurcation of iliac arteries (planes **a**, **b**, **c** and transverse panel **e**). The celiac artery, SMA and rt. renal artery emerged apparently from the true aortic space while, the left renal artery seemed atrophic and emerged from the false lumen (panel **d**). The inferior mesenteric artery (IMA) emerged from the false lumen (plane **c**). The aortic artery diameters were within the normal ranges along 8 years of follow-up. The iliac arteries looked normal and also the perfusion of the splanchnic system and lower extremities. In the last CTA study (2011), in addition to the old findings, a new finding (Panels **f**, **g**) of a dissection of the innominate artery (**f**) continuing into the right subclavian artery (**g**), with no evidence for dissection in the right carotid artery, was demonstrated

This group comprised 6 patients, V-2, VI-23, VI-26, VI-27, VII-1, and VII-3, with extensive arterial dissections involving many arteries. On presentation, these family members complained of dyspnea and sudden onset of severe pain in the chest, abdomen, or upper and lower extremities (Table 1). Diagnosis was made in 4 patients, within three hours after their admission to the hospital; for the remaining two patients the diagnosis was made two and eight days after their admission. All were diagnosed with aortic dissections, 5 involving the ascending aorta extending to the descending aorta (Stanford classification type A dissections) and one involving just the descending aorta (type B dissection) (Table 2). None of the patients had unequivocal evidence of an aortic aneurysm at the time of the dissection. An aneurysm of subclavian artery was identified in three patients, VII-26 and VII-27, who were sisters, and VII-3, and an aneurysm in the innominate and carotid arteries was found in two patients, VII-27 and VII-3. Patient VI-26 had a dilatation of the aortic arch between the left subclavian and carotid arteries (Table 2, Fig. 2).

The outcome of this group was poor. Five of the 6 patients died shortly after presentation and diagnosis. Patients V-2, VII-1, and VI-27 died within three weeks after their admission to hospital. Emergent surgical repair of the ascending aorta was done on patients, VI-23, VI-27, and VII-3. The repairs were successful in patients VII-3 and VI-23 and they were discharged after four and six weeks, respectively, to a rehabilitation center. These two patients were re-admitted after four months (patient VII-3) and six months (patient VI-23) because of extensive vascular dissection of the abdominal aorta, and renal and superior mesenteric arteries and multi-organ failure, and they died within 48 h of their admission. TAAD and AAAD were diagnosed in patient V-2 on post-mortem examination. Patient VII-1 was initially diagnosed as having a mesenteric vascular embolus and underwent surgery for a segmental bowel resection. During the surgery, a abdominal aortic dissection was found, leading to bowel infarction. The attending surgeons were not able to complete an endovascular graft repair of the aorta and the patient died due rapid hemodynamic deterioration. Patient VII-26 is the only surviving patient of Group 1. She is currently 52 years of age. 8 Years after presenting with the type 3B aortic dissection, she had an extension involving the left renal artery, which arose from a false aortic lumen and resulted in a dysfunctional left kidney (Fig. 2d). Since she was hemodynamically stable at the time of diagnosis, she has been monitored by regular echocardiography and CTA and treated with an angiotensin converting enzyme inhibitor and furosemide and has been stable ten years. The siblings and close relatives of patients VII-26 and VII-27 were interviewed and their medical files reviewed. Our investigation revealed that both patients (VII-26 and VII-27) had complained of bilateral arm weakness, effort-induced shoulder pain, and general fatigue. The shoulder pain was induced by minor physical activity, such as raising their hands above the head, as done in hair dressing, and while doing housekeeping chores.

In the absence of a definitive diagnosis on post-mortem examination, we hypothesize that the death of individual VI-28, a 35-year-old brother of patients VI-26 and VI-27 and the son of the patient V-5, was due to rupture of an aortic dissection that either followed or caused a minor road accident.

### Group II patients

This group comprised three patients, V-15, V-22, and VI-24, who presented with either TAA and aortic dissection or both confirmed by echocardiography and CTA. These individuals presented later in life than Group 1, in the sixth or seventh decade. In these cases, the diagnosis was made when the individual presented with acute chest pain, effort-induced breathlessness, and weakness. Patients V-22 and VI-24 had type-2 diabetes mellitus and TAA and TAAD were diagnosed after they were hospitalized with acute ischemic heart disease. Review of the medical history and the absence of a definitive diagnosis in the post-mortem reports, we suspect that three family members, IV-5, IV-7 and IV-8, who were either the parents or grandparents of the group I and group II patients, died because of complications of dissections. A few days before their deaths, these individuals all complained of chest and abdominal pain.

### Whole-genome genotyping and sequencing of the MYLK gene

**Fig. 3:**
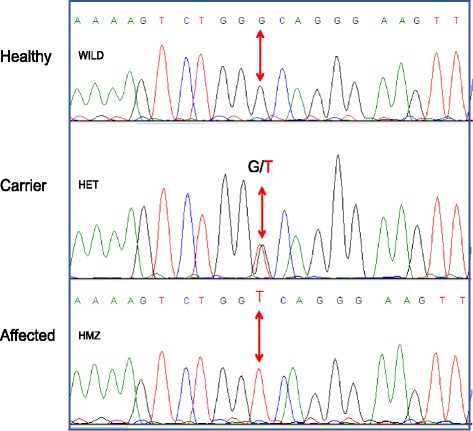
The MYLK gene novel missense mutation, c.4471G > T, Ala1491Ser, located in exon 27. Her we show the electropherogram of a healthy control, carrier and homozygote representative patient (VI-26). This change converts a codon for alanine (GCA) to a codon for serine (TCA)

Since the mode of inheritance of familial TAAD is usually autosomal dominant [12], we posited that the six group I patients are homozygous for the familial mutation, and that this mutation was the underlying cause for the phenotype of these patients. Accordingly, we used the DNA of patients VII-3 and VI-26 to perform single nucleotide polymorphisms (SNPs) arrays and regions of shared homozygosity in their genomes were identified. We found an 18 Mb homozygous region on chromosome 3 (3q21.1), which harbors *MYLK*, a gene known to predispose to aortic dissection without aneurysm [9, 12]. Consequently, we sequenced the coding exons and their flanking regions of *MYLK* using the DNA of patient VI-26 (Fig. 3). We identified a missense mutation, c.4471G > T, Ala1491Ser, in exon 27 *MYLK.* This change was not found in the ClinVar, HGMD, Exome Aggregation Consortium (ExAC) and the 1000 genomes (http://www.1000genomes.org) databases. The mutation was not detected in the DNA of 100 healthy individuals of the same ethnic background. A BLAST comparison of the amino acid sequence of orthologous MLCK proteins from 35 different species revealed that Ala1491 is conserved (data not shown).

Moreover, we performed the segregation and LOD score calculation studies and for the disease model we used was additive with a penetrance of 0.3 for one copy of the mutation, a penetrance of 0.9 of two copies of the mutation, and using a disease prevalence of 0.001. The data consists of measuring the MYLK gene, and marking the results by Homozygous, Hetrozygous, and Wild. Exact two-point LOD score of 3.02 was computed using the Superlink-Online system [17].

### Effect of MYLK p. Ala1491Ser on myosin light chain kinase activity

The *MYLK* variant, Ala1491Ser, falls in the kinase domain of the protein. Kinase activity of the Ala1491Ser protein showed a significant reduction in kinase activity compared to wildtype but no further decrease based on calcium/calmodulin activation (Fig. 5). Thus, the MYLK pathogenic variant does disrupt kinase activity but to a lesser degree than previously reported mutations causing autosomal dominant thoracic aortic disease [12].

### Histologic studies

**Fig. 4:**
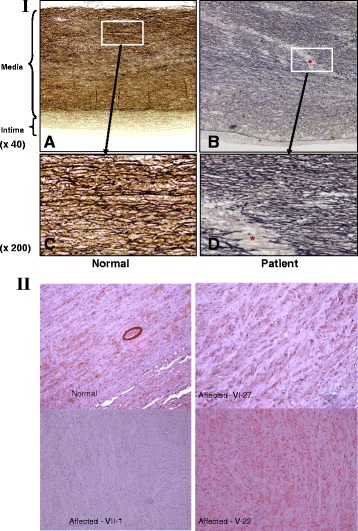
(panels 4-I; 4-II): Pathologic aorta studies performed in 8 patients (5 from group-1 and 3 from group-2) diagnosed with aortic dissection involving the ascending, thoracic and abdominal aorta, as were described in three medical centers in the north of Israel. In brief, “cystic medial degeneration and necrosis” was the main common pathological finding reported in all patients. This was accompanied with more details such like “focal fibrin deposits admixed with neutrophils with signs of cystic medial necrosis” “adventitial and periadventitial fibrosis”. Here, we compare between normal and affected aorta sections from patient VI-27, VII-1 and V-22 (Fig. 1) by using different aorta staining. First, a reprehensive Elastica – van Gieson staining presented in the upper panels (4-I): **a** and **c** (for normal aorta) showing the regular layered pattern of elastic tissue as expected in normal media, this was compared with affected aorta samples (panels **b** and **d** patient VI-27) showing disruption and fragmentation of elastic lamellae of the aortic media with formation areas devoid of elastin (asterisk) that resemble cystic spaces. Lower panels (4-II) show MLKC staining Immunohistochemistry. We used primary antibody directed against human MLCK in all aortic sections obtained from normal and three patients namely VI-27, VII-1 and V-22 (Fig. 1). The panels represent staining of normal aorta section (normal) where the MLCK protein is stained brown (encircled) compared with aorta staining in three patients’ sections. The MLCK is not detected in patients VI-27, VII-1 (both found homozygous to the Ala1491Ser mutation) while, it is weak but diffuse in patient V-22 (heterozygous for Ala1491Ser mutation)

Medial necrosis was seen in all of the aortic samples from the living and deceased patients in whom an emergency endovascular repair was performed as seen in elastica – van Gieson aorta staining (Fig. 4).

Immunohistochemical staining for MLCK: Fig. 4-II, displays representative images of aortic specimens for patients VI-27, VII-1 and V-22 that were immunostained for MLCK. MLCK was barely not detected (score 0–1 for both intensity and extent) in the medial layer of the aorta from patients VI-27, VII-1 and was weak but diffuse (score 3) in patient V-22, compared with normal staining (score 6) as seen in normal aortic specimen.

### Phenotype-genotype correlations

We then investigated whether a genotype-phenotype correlation exists in the five living patients and all living family members, and found that the c.4471G > T transversion segregated and adequately matched the clinical phenotypes of the 45 tested individuals. Specifically, three of the group I patients, VII-3, VI-26, and VI-27, were homozygous for the c.4471G > T mutation and their parents were heterozygous. The DNA that was extracted from the paraffin-embedded specimen of patient VII-1 had degraded and did not meet the quality requirements for Sanger sequencing analysis. We could not test the DNA of deceased patient VI-23 because there were no specimens for DNA extraction. When we tested the parents, V-20 and V-21, of patient VI-23, we found that both were heterozygous for the mutated allele. Accordingly, we inferred that the patient VI-23 was homozygous for the mutation because the clinical phenotype was well correlated with that of the group I patients (Table 1). We found 22 individuals were heterozygous for the mutant allele. Two of these individuals, V-22 and VI-24, were group II patients, and had successfully undergone reconstructive surgery for TAA and TAAD. One young asymptomatic carrier, VI-1, was of special interest because he was the son of the first documented patient in the family, V-2, who was diagnosed as having familial TAAD after analyzing specimens that were collected from the post-mortem examination. The carrier status of invidual VI-1 provides a strong evidence that patient V-2 was probably homozygous for the family mutation and his wife, V-3, most likely has the wildtype alleles. Twenty heterozygous carriers of the *MYLK* mutant allele (average age 45-years-old) were investigated for aortic dilatation using echocardiography. The diameters of the aortic root and aortic artery of 18 carriers were within the normal range (less than 4 cm, and 3.5 cm, respectively). In individuals V-7 and V-9 (brothers of patient V-2), the diameters of the aortic root (3.9 and 3.8 cm) and ascending aorta (3.4 cm/m^2^ for both). Diameters of the the descending aorta of the two were outside the normal range (4.5 cm and 4.7 cm, respectively).

Four family members, IV-5, IV-7, IV-8, and V-15 (Fig. 1), died suddenly when they were in the six or seventh decade of their life. Aortic dissection was documented only in patient V-15, ten days after admission to hospital and before any surgical intervention could be done. We surmise that this patient was heterozygous for the mutated allele because three of his offsprings, VI-9, VI-12, and VI-13, are heterozygous while the other tested offsprings VI-5, VI-10, and VI-11 were homozygous for the wild allele. We also surmise that the family member IV-5, whose wife, IV-6, who is unrelated, is heterozygous for the mutated allele because his son, V-15, died of TAAD and the daughter, IV-16, and the granddaughter, VI-14, are both heterozygous for the mutated allele. The mother, VI-14, of the two deceased homozygous patients, VII-1 and VII-3, is also heterozygous for the mutated allele.

## Discussion

**Fig. 5:**
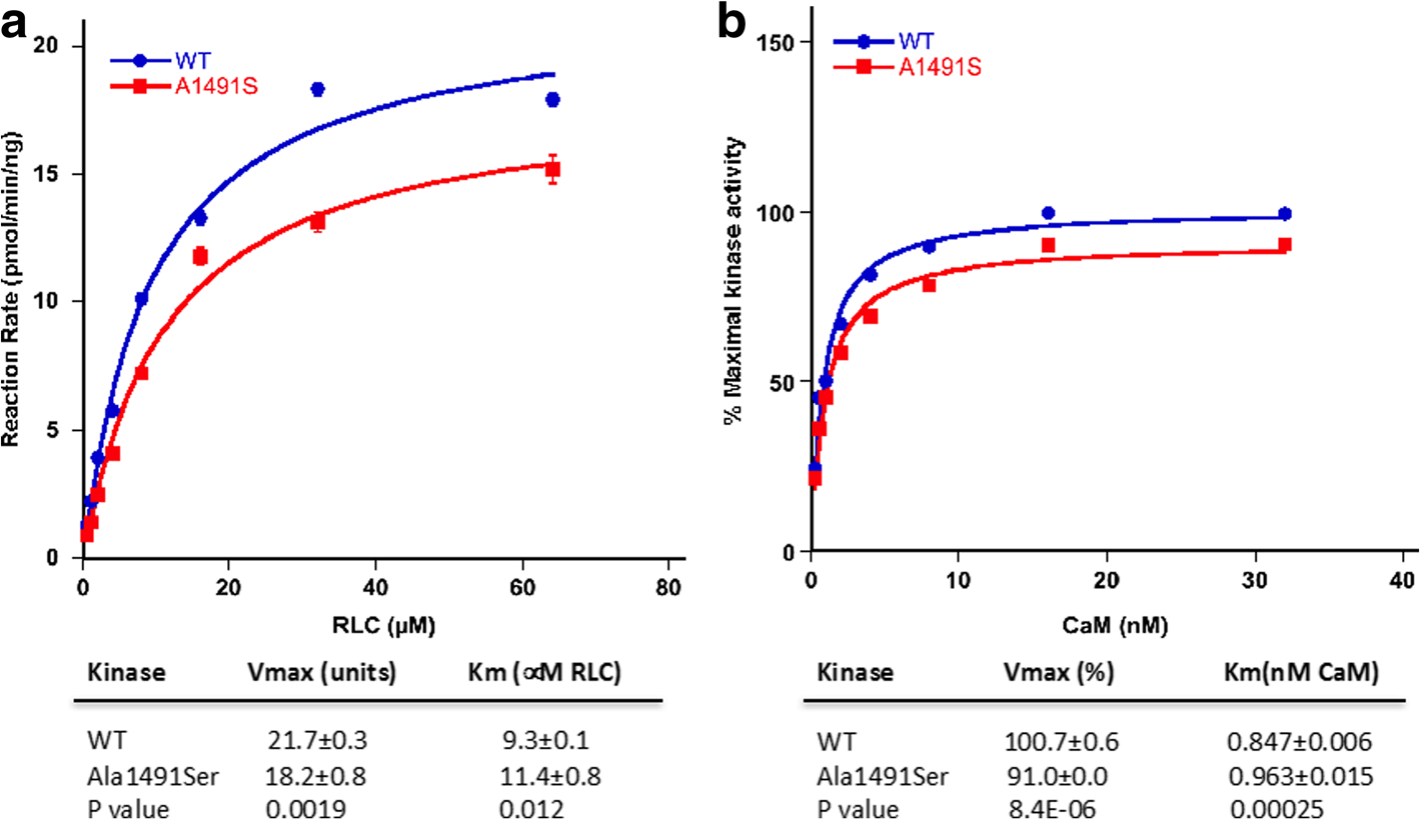
Kinase activity of the *MYLK* wild-type (WT) and Ala1491Ser proteins. HeLa cells were transfected with constructs of the WT and mutant MLCK. **a**. the rate of ^32^P incorporation into the regulatory light chain (RLC) was measured. The maximal activities of WT (blue circles) and Ala1491Ser (red squares) were obtained at different RLC concentrations. **b**. Calmodulin (CaM) activation of WT and mutant MLCK proteins. The relative precentage of maximal kinase activity of WT (blue circles) and Ala1491Ser (red squares) was plotted versus various CaM concentrations. The data points represent the mean ± standard error of three or more determinations. The data were fit to the Michaelis-Menten equation for calculation of the V_max_ and K_m_ values

In this report, we describe a novel variant (p. Ala1491Ser, c.4471G > T) in *MYLK* in a large, inbreed family. As described in the myosin light chain kinase activity assay (Fig. 5), the MYLK p. Ala1491Ser pathogenic variant does disrupt kinase activity but to a lesser degree than previously reported mutations causing autosomal dominant thoracic aortic disease [12]. Clinically, this mutation presented as an aortic aneurysm and dissection of the thoracic and/or abdominal aorta and the subclavian, innominate, renal, superior mesenteric and iliac arteries in two distinct phenotypes that are correlated with homozygosity or heterozygosity for the *MYLK* variant.

Since we found that the group-I patients were homozygous for the c.4471G > T mutation (p. Ala1491Ser) and the amount of MLCK in the aortic specimens was reduced, we concluded that the mutation accounted for the phenotype of this group of patients. Moreover, family’s pedigree and mutation segregation analysis, supported the autosomal recessive mode of inheritance in group-I or dose dependent autosomal dominant with 100% penetrance in homozygous patients. Since we also found that the group-II patients were heterozygous for the mutation, we concluded that the mutation accounted for the phenotype of this group of patients. However, the most appropriate mode of inheritance of the phenotype of this group is autosomal dominant with incomplete penetrance.

Six disease-causing mutations (c.5275 T > C, p.S1759P; c.5260G > A, p.A1754T; c.3637G > A, p.V1213 M; c.4195G > A, p.E1399K; c.4438C > T, p.G1480X [12]; and c3272_3273del, p.S1091X) in *MYLK* have been previously confirmed to cause familial TAAD [12, 14]. Two mutations are truncating mutations (p. Arg1480X, c.4438C > T and c3272_3273del, p.Ser1091*) predicted to lead to nonsense mediated decay and haploinsuffiency. Patients who carry these mutations present with acute aortic dissection with little to no enlargement of the aorta [12]. This phenotypic expression of the truncating mutation of the *MYLK* gene differs from that of the missense mutation that we found in this investigation in that the homozygous and heterozygous carrier patients develop arterial aneurysms and dissections. The group I and II phenotype discrepancy can be well explained by the mutation dose dependent disease behavior. We already describe a resembling type of inheritance in myotonia congenita, where the homozygous mutated patients were severely affected and their muscle myotonic contractions started in the first decade of life [18].

It should be noted that a comprehensive family medical history and clinical data analysis of group-I patients (hmz for p. Ala1491Ser mutation) had excluded congenital bladder or intestine involvement. Thus, in our large kindred we determine that there was no evidence for congenital Megacystis Microcolon Intestinal Hypoperistalsis Syndrome (MMIHS) which is characterized by loss of smooth muscle contraction in the bladder and intestine [15].

Of note the pathogenic variants found in MMIHS patients was a homozygous truncating mutation, c.3838_3844dupGAAAGCG [p.Glu1282_Glyfs∗51, in the first family and splice-site variant (c.3985 + 5C > A), in the second family [15]. The first mutation is located in the Ig-like C2 type-8 within the MLCK protein actin (calcium/calmodulin sensitive) binding domain [12] while the second mutation is affecting the proper RNA splicing and expression studies and splicing assays indicated that both variants affect normal MYLK expression [15]. We assume that MYLK gene null mutation can lead to MMIHS in homozygous state.

Finally, our finding of a missense mutation of the *MYLK* gene in a large consanguineous family with aneurysmal disease may provide an excellent opportunity to conduct a long-term prospective study to determine the disease early signs and to clarify the contribution of new genetic markers such like the microRNAs (e.g miR-195 and miR-29b) [19] to the family disease in homozygous and heterozygous carriers and non-carriers of the mutation as part of a prediction and prevention strategy for this family.

## Conclusions

We found that MYLK gene Ala1491Ser mutation affect the kinase activity and clinically, it presents with vascular aneurysms and dissection. We describe a distinct genotype phenotype correlation where; heterozygous patients have mild late onset and incomplete penetrance disease compared with the early onset severe and generally fatal outcome in homozygous patients.

## Abbreviations

MLCK: Myosin light chain kinase
TAAD: Thoracic aortic aneurysms and aortic dissections
